# Burnout and its Associated Factors in Medical Students of Lahore, Pakistan

**DOI:** 10.7759/cureus.390

**Published:** 2015-11-29

**Authors:** Yumna Muzafar, Hibbah H Khan, Huma Ashraf, Waqas Hussain, Hifsa Sajid, Marium Tahir, Abdul Rehman, Aleena Sohail, Ahmed Waqas, Waqas Ahmad

**Affiliations:** 1 Medical Student, CMH Lahore Medical College and Institute of Dentistry, Lahore Cantt, Pakistan; 2 Medical Student, Allama Iqbal Medical College, Lahore, Pakistan; 3 Final year MBBS Student, CMH Lahore Medical College and Institute of Dentistry, Lahore Cantt, Pakistan

**Keywords:** medical students, burnout, lahore, associated factors, pakistan, Academic Performance

## Abstract

Introduction:

Burnout is a widely known phenomenon. It is defined as a state of prolonged physical and psychological exhaustion and is experienced virtually by every medical student due to the highly demanding nature of medical education. This study probes into the prevalence and psychosocial determinants of burnout in Pakistani medical students.

Methods:

A descriptive, cross-sectional study design and convenience (non-probability) sampling technique were employed in undergraduate medical students from years 1-5. A total of 777 medical students from two medical colleges were included in the study from May-August, 2014. An English version of the Copenhagen Burnout Inventory (CBI) and a series of demographic questions, intermixed with questions from other topics, were included in the questionnaire. Data was analysed by using SPSS ver.21.

Results:

The majority of students were females and enrolled in the third year of MBBS. Of the medical students involved, 30.6% were found to have high/very high levels of burnout (Kristenson’s burnout scoring). Although 38.7% of students said that they did not feel burned out after reading the definition of burnout given in the questionnaire, 35.9% out of these students actually had high levels of burnout according to CBI. According to the multiple regression analysis, burnout in medical students was significantly associated with age, gender, doctor parents, no help or no supportive resources (e.g., from colleagues), lack of time off, lack of belief in what you do, fear of big consequences of failure, family responsibilities, and uncertain future. Perception of teachers lacking leadership skills and doing too much study with little balance was associated with low burnout scores.

Conclusion:

There is a high prevalence of burnout in Pakistani medical students. The present study identifies several factors associated with burnout in Pakistani medical students. Although these factors are a part of daily life of medical students, their identification should prompt the use of effective coping strategies and skills, thus, minimising their burnout levels.

## Introduction

In the fast-paced, competitive world of today, many people consider stress to be a part of life. Increasingly high demands of the educational system on students mean that they, too, are not immune to emotional strain and anxiety. Undergraduate medical education, in particular, is notorious for being long and emotionally taxing. Medical students often encounter intense and demanding circumstances in the course of their academic studies. This leads to reports of high levels of stress and psychiatric morbidity in them, including, but not limited to, depression and GAD (General Anxiety Disorders) [1-3]. In a cross-sectional questionnaire-based study conducted at Surat Medical College, Pakistan in 2012 by Solanky, et al., almost all the medical students reported stressor experiences of which 40% were severe in nature while another study conducted in found nearly half the students in medical school to be anxious and/or depressed [2-3]. Given these taxing circumstances, it is only predictable that at some point a large number of these students would be burnt-out.

First coined in the 1970s by the American psychologist Herbert Freudenberger, the term “burnout” is broadly described as a state of chronic stress leading to physical and emotional exhaustion, cynicism, detachment, feelings of inadequacy, and lack of accomplishment [4]. Despite the existence of multiple similar definitions, no single interpretation has been agreed upon so far. Freudenberger defined burnout as “the extinction of motivation or incentive, especially where one’s devotion to a cause or relationship fails to produce the desired results” and used it to describe the consequences of severe stress and high standards experienced by people working in "helping" professions, such as doctors and nurses, who aid others at a cost to themselves [5]. Today, however, “burnout” has become a popular term and is used not only for illustrating the dark side of self-sacrifice but rather as a disorder that seems to affect anyone, from stressed-out professionals, students, and celebrities to over-burdened employees and homemakers. Despite this popularity, burnout is not yet recognized as a psychiatric disorder in its own right in the DSM-5 (Diagnostic and Statistical Manual of Mental Disorders, 5th Edition). This lack of universal acceptance means that many do not believe in burnout as a primary diagnosis due to its close resemblance to depressive disorders and alleged ambiguity in the inventories currently used for assessing burnout in individuals [6-7].

A study published in 2014 based on a national survey carried out in the US found that medical students, residents/fellows, and physicians with less than five years' practice were more likely to be burnt-out compared to the general population [8]. A 2013 literature review by Ishak, et al. revealed that burnout is prevalent during medical school, with major US multi-institutional studies estimating that at least half of all medical students may be affected by burnout during their medical education. Further, it illustrated that burnout may persist beyond medical school and is, at times, associated with psychiatric disorders and suicidal ideation [9].

Over the years, quite a few studies have attempted to pinpoint the factors that lead to burnout. An assessment of burnout in British medical students revealed that the year of study, physical activity, and smoking status significantly predicted the emotional exhaustion component of the burnout inventory whilst gender, year of study, and institution significantly predicted depersonalization. Alcohol binge score, year of study, gender, and physical activity significantly predicted personal accomplishment [10]. Another study tested the connection between burnout and personality type, demonstrating that impulsivity trait is associated with high levels of burnout with neuroticism and Type-A behavior also showing some association. Apart from these, depressive symptoms and financial concerns in the first year also displayed high association with burnout in later years [1]. Yet another study found the prevalence of burnout to be higher among those who did not have confidence in their clinical skills, those who felt uncomfortable with course activities, and those who did not see the coursework as a source of pleasure [11]. The effect of gender on the possibility of burnout is rather ambiguous with literature that shows equal rates of burnout in both genders and literature that demonstrates higher rates of burnout in females, although the general stress levels are found to be higher in females compared to males [12-13]. Also related to the risk of burnout are recent negative events in a students' personal life [14].

The goal of imparting medical education is to produce knowledgeable, skillful, and professional physicians. As is evident, however, students' mental and emotional health is adversely affected in medical school, which results in both personal and professional deterioration including, but not limited to substance abuse, relationship problems, suicidal ideation, worsening relationship with faculty and lack of empathy and professionalism [15-16].

Medical students worldwide encounter tremendously challenging conditions and feelings of inadequacy, which are understandable in a field that deals with human life. The cultural and social dynamics of Pakistan create unique stresses in their lives. This study probes into the determinants and prevalence of burnout in Pakistani medical students, which is a subject that has not, to our knowledge, been adequately scrutinized previously.

## Materials and methods

### Study design

Descriptive, cross-sectional study design and convenience (non-probability) sampling technique was employed. In Pakistan, undergraduate medical education lasts five years. This includes two pre-clinical years and three clinical years. The prevalence of burnout in medical students of academic year one to five from two medical colleges, a government college (Services Institute of Medical Sciences, SIMS) and a private college (CMH Lahore Medical College), were assessed between May 2014 and August 2014. The English version of the Copenhagen Burnout Inventory (CBI) and a series of demographic questions were included in the questionnaire. The minimum sample size required for the present study was calculated as 384 with a 95% confidence level and a confidence interval of five.

### Ethics statement

The CMH Lahore Medical College and Institute of Dentistry Ethics Review Committee approved the study. Participants who agreed to participate were explained the nature and the objectives of the study, and informed consent was obtained.

### Instrument

The Copenhagen Burnout Inventory was employed, which measures three aspects of burnout in respondents' lives: personal, work-related, and patient-related. The present study assesses only the personal aspects of burnout, thus, employing the personal burnout scale consisting of six items. The Cronbach´s alpha for the personal burnout scale is 0.87 which reflects a good internal consistency of this scale. All burnout items were intermixed. In this study, responses were made in the following categories: Always, Often, Sometimes, Seldom, Never/Almost Never with each corresponding to a score 100, 75, 50, 25, 0. Total score on the scale is the average of the scores on the items [17].

### Data analysis

IBM SPSS Statistics for Windows, version 21.0 software (IBM Corp., Armonk, NY, USA) was used for analysis. Frequencies were calculated for demographic variables. Mean burnout scores were calculated and divided into quartiles. They were then further recoded as a dichotomous variable (cut-off value = 50). Burnout scores less than 50 were coded as low levels and > 50 as high burnout levels.

The frequencies of stressors were grouped into dichotomies as follows: never/rarely/sometimes and often/always. Multiple regression analysis (backward method) was run to identify the significant determinants of scores on the Copenhagen Burnout Inventory. The burnout scores were used as a dependent variable and age, gender, year of study, residence, the profession of parents (doctors/ not doctors), and various stressors associated with medical school were entered as predictor variables. 

## Results

### Demographic characteristics

A total of 777 medical students participated in the survey. Out of these, 447 (57.5%) were female and 330 (42.5%) were male students. Most of the students belonged to third year MBBS (266, 34.2%) followed by first^ ^year (184, 23.7%), second^ ^year (132, 17%), fifth year (105, 13.5%), and fourth year (90, 11.6%). Most of the students were Pakistanis (738, 95%) while the remainder had a foreign background (39, 5%). Almost 244 (31.4%) of the students had physician parents. According to Kristenson’s criteria of burn out levels, most of the students had low burn out levels 328 (42.2%), nil 81 (10.4%), moderate 123 (15.8%), high 156 (20.1%), and very high 89 (11.5%) [18]. Chi-square associations of demographics and burnout levels are given in Table 1.

**Table 1:** Association between demographics of students and burn out levels (n= 777) Chi-square= X^2^

	Burnout Levels			
Variables	Low levels	High levels		
	Frequency (n)	Frequency (n)	χ² value	p-value
Gender			18.13	< .001
Male	203 (61.5%)	127 (38.5%)		
Female	206 (46.1%)	241 (53.9%)		
Study year			.40	.56
Pre-clinical group	162 (51.3%)	154 (48.7%)		
Clinical group	247 (52.6%)	214 (46.4%)		
Doctor parents			.01	.5
Yes	128 (52.5%)	116 (47.5%)		
No	281 (52.7%)	252 (47.3%)		
Background			4.6	.03
Pakistani	395 (53.5%)	343 (46.5%)		
Foreign	14 (35.9%)	25 (64.1%)		
Residence			2.3	.08
Off campus	209 (50.1%)	208 (49.9%)		
On campus	200 (55.6%)	160 (44.4%)		
Relationship			5.9	.12
Single	338 (54.8%)	279 (45.2%)		
Married	24 (48%)	26 (52%)		
In a relationship	30 (42.3%)	41 (57.7%)		
Engaged	17 (43.6%)	22 (56.4%)		
Current rotation			1.9	.38
Outpatient	45 (47.9%)	49 (52.1%)		
Ward	202 (55%)	165 (45%)		
Not applicable	162 (51.3%)	154 (48.7%)		
Do you feel burnt out?			25.97	< .001
No	193 (64.1%)	108 (35.9%)		
Yes	216 (45.4%)	260 (54.6%)		

According to the Chi-square analysis, day scholars, female gender, and students with a foreign background reported a higher incidence of burnout. After giving the definition of burnout, "burnout is a state of prolonged physical and psychological exhaustion"[17], students were asked “Do you feel burnt out?". Out of 777 students, 301 (38.7%) students reported negatively to the question and 35.9% (108/301) of these students, in fact, had high levels of burnout on CBI (p < 0.01).

### Stressors

Stressors most frequently cited by the respondents were: too much study with little balance, lack of time for recreation, lack of sleep, lack of time off, high frequency of tests, fear of failure, insufficient rewards of acknowledgment of your work, sense of never ending competition, scoring lower than hoped for, and high parental expectations. Detailed results are given in Table 2.

**Table 2:** Frequency of stressors as reported by the respondents (n = 777)

Stressors	Rare (Percentage)	Frequent (Percentage)
Too much study with little balance	453 (58.30)	324 (41.70)
No supportive colleagues	529 (68.08)	248 (31.92)
Too little social support	540 (69.50)	237 (30.50)
Lack of time for recreation	455(58.56)	322(41.44)
Lack of sleep	458 (58.94)	319 (41.06)
Lack of time off	426 (54.83)	351 (45.17)
Lack of supportive relationships	530 (68.21)	247 (31.79)
Accommodation away from home	538 (69.24)	239 (30.76)
Lack of belief in what you do	581 (74.77)	196 (25.23)
Choosing field of medicine against your interest or family pressure	596 (76.71)	181 (23.29)
High frequency of tests	416 (53.54)	361 (46.46)
Fear of failure	475 (61.13)	302 (38.87)
Insufficient rewards of acknowledgment of your work	447 (57.53)	330 (42.47)
Poor communication with colleagues and teachers	511 (65.77)	266 (34.23)
Poor leadership skills of teachers	521 (67.05)	256 (32.95)
Gender discrimination in college	616 (79.28)	161 (20.72)
Discrimination based on social role (wife, guardian of household)	636 (81.85)	141 (18.15)
Discrimination based on caste	672 (86.49)	105 (13.51)
Sense of never ending competition	473 (60.88)	304 (39.12)
Scoring lower than hoped for	472 (60.75)	305 (39.25)
Family responsibilities	592 (76.19)	185 (23.81)
Uncertain future	534 (68.73)	243 (31.27)
Loss of loved one in past 12 months	650 (83.66)	127 (16.34)
Witnessed major illness of close family member	623 (80.18)	154 (19.82)
High parental expectations	476 (61.26)	301 (38.74)
Financial constraints	615 (79.15)	162 (20.85)

### Determinants of high burnout levels

According to multiple regression analysis, high burnout levels were significantly associated with age, gender, physician parents, no help or no supportive resources (e.g. from colleagues), lack of time off, lack of belief in what you do, fear of the big consequences of failure, family responsibilities, and uncertain future. Students who perceived that their teachers lacked leadership skills and were doing too much study with little balance had low burn out scores. Detailed results have been presented in Table 3.

**Table 3:** Multiple regression model for burn out scores (n = 777) ANOVA F= 12.5, P < .005, Adjusted R2= 14.0, backward method

Predictor	B	Std. error B	Beta	P- value
(Constant)	-26.437	58.324		.650
Age	4.372	2.356	.062	.064
Gender	41.440	8.085	.172	.000
Doctor parents	38.096	18.312	.070	.038
Too much study with little balance	21.333	11.085	-.089	.055
No help or no supportive resources (e.g. from colleagues)	32.293	11.026	.127	.004
Lack of time off	24.538	10.700	.103	.022
Lack of belief in what you do	23.256	10.721	.085	.030
Fear of the big consequences of failure	24.497	11.019	.101	.027
Poor leadership skills of teachers	-19.490	10.483	-.077	.063
Family responsibilities	22.795	11.670	.082	.051
Uncertain future	22.105	11.225	.086	.049

## Discussion

In our study, about half the medical students (47%) met the criteria for moderate, high, or very high levels of burnout. This matches well with several studies on levels of burnout among medical students. For instance, in a study of Swedish medical students, Dahlin, et al. found high levels of burnout in 47% of the students [1]. Similarly, Dyrbye, et al. found a burnout incidence of 45% among the medical students of Minnesota [14]. Mazurkiewicz, et al., however, reported higher levels of burnout (71%) in third-year medical students of Mount Sinai School of Medicine, New York [19].

An interesting finding in our study was the fact that many medical students were unaware of their own burnout. Despite answering negatively to the question “Do you feel burnt out?”, 36% of the students were actually found to be suffering from high levels of burnout based on their responses to CBI. This lack of awareness is alarming since it can lead to neglect of symptoms and progression to greater levels of burnout. Numerous studies have identified the positive effects of self-awareness-based interventions in alleviating stress among both medical students and physicians. For instance, Shapiro, et al. found mindfulness-based stress reduction to reduce both depression and anxiety among medical students [20]. Similarly, Krasner, et al. found that self-awareness exercises among primary care physicians reduced both burnout and mood disturbance in them and improved their empathy [21]. We suggest promoting self-awareness strategies among medical students. Simply making them aware of their burnout could go a long way in enabling them to cope with it.

Our study identified numerous stressors among medical students, most of which were academic in nature. These stressors, including lack of sleep, lack of time off, high frequency of tests, fear of failure, and high parental expectations among others, correlate with those in published literature on the subject. For instance, in a study of stressors among Pakistani medical students, Shah, et al. highlighted high parental expectations, frequency of examination, worrying about the future, sleeping difficulties, and performance in periodic examinations as the common stressors, which are quite similar to the stressors of our study [22]. Similarly, Sreeramareddy, et al. found the vastness of syllabus, tests/exams, high parental expectations, and lack of time and facilities for entertainment as major stressors in undergraduate Nepalese medical students, again concurring with our results [23]. Waqas, et al. found that most of the Pakistani medical students (77%) of their study were poor sleepers and that their lack of proper sleep contributed to high-stress levels among them, supporting our result of lack of sleep as a common stressor [24].

Age was found to be significantly associated with burnout. Older medical students (in senior medical years) were more burnt out than younger students (in junior medical years). This is in accordance with Dyrbye, et al.’s study, which shows that senior medical years are associated with greater burnout [14]. A study by Shaikh, et al. also supports our results. They found that fourth and fifth-year medical students had the greatest stress as compared to junior medical students [25].

We found female gender to be significantly associated with high burnout levels. Studies on burnout of medical students generally agree with our results. For instance, a study by Backović, et al. on final year medical students of Serbia found female students to be significantly more stressed and burnt out as compared to male students [12]. Another study by Dahlin, et al. also found female medical students to be more exhausted than their male counterparts [1]. The higher burnout among female medical students is more likely due to a higher perceived impact of stressors rather than a higher number of stressors as compared to male students. This view is in accordance with Ranjita, et al.’s study on the academic stress of college students in which female students rated negative events more often and more markedly as compared to male students showing an increased emotional response to stressors [26]. Accordingly, Backović, et al. reported that female medical students declared high-stress effects from contact with patients and autopsy more frequently than male students [12]. Thus, the stressors of medical school appear to impact female students more severely and, as a consequence, cause more frequent burnout in them.

Our study found significantly greater burnout among medical students whose parents were doctors. This finding is in agreement with Sreeramareddy, et al. (2007) who found greater stress among medical students whose parents were medical doctors [23]. One possible explanation for this is the fact that medical doctors have perfectionistic attitudes [27]. It is likely that their perfectionism extends to their parenting as well and results in increased expectations from their children. It is well-known that perfectionistic expectations of parents can lead to both increased depression and dysfunctional behaviour among their offspring [28]. Thus, the higher expectations of their parents and increased stress of meeting these expectations may be responsible for greater burnout among medical students with doctor parents. Another explanation for this relationship could be the hereditary nature of perfectionism. As pointed out by Soenens, et al., both maladaptive and adaptive perfectionism in parents can be transferred to their children because such parents employ greater psychological control in parenting [29]. The association of perfectionism with stress is well-known [30]. Therefore, the perfectionism of doctor parents passed down to their children could be responsible for greater burnout among such medical students.  

Our study found an interesting factor associated with burnout, which is a lack of belief in the medical profession. The fact that even individuals training to become doctors do not believe in the medical profession is quite appalling. A review of medical literature shows that such doubts about the medical profession are common not only among the general population but also among physicians [31-32]. In a special report, Zuger points out that many physicians are dissatisfied with the medical profession in its current state and that this dissatisfaction is responsible for increased psychological morbidity of physicians on one hand and poor clinical management and substandard medical care on the other [32]. She goes so far as to liken the prevailing emotional climate among medical professionals to “the atmosphere surrounding a deathbed”. In this context, our finding makes perfect sense. Thus, medical students harbouring doubts and dissatisfaction about their profession are more likely to experience psychological distress and burnout as compared to those who don’t harbour such doubts. Ahmad, et al. found out in a study that medical students of Pakistan have the most doctor-centered attitudes in the entire globe, which could be attributed to the fact that medical students don’t believe in their profession and are burnt out to the extent that they do not care for the patient’s rights [33].

Another cause of burnout identified by our study is family responsibilities. The high commitment required for medical education can predictably add up with the family demands to increase stress on medical students. Both may even clash at times as found by Clark, et al.’s study according to which the stress of medical education can often cause difficulties in personal relationships [34]. In Pakistan, married medical students and those belonging to poor families are expected to support their families financially. Such medical students may find it difficult to meet the demands of medical education on the one hand and the financial and emotional support of their families on the other. Thus, the combined demands of medical education, family, and the stress arising from occasional conflicts between these demands can exhaust the students to the level that they become burnt out.

Among academic factors, lack of time off, fear of big consequences of failure, lack of help or supportive resources, and uncertain future were significantly associated with burnout among medical students of our study. As already mentioned above in the discussion of stressors, both Shah, et al. and Sreeramareddy, et al. have highlighted many of these factors as common stressors among medical students [22-23]. Dahlin, et al., in a study of Swedish medical students, cited both “worries about future competence/endurance” and “non-supportive climate” as major causes of stress among medical students [35]. Since these factors are repeatedly mentioned in literature as stressors, it is hardly surprising that they should cause burnout in medical students who are chronically exposed to these factors.

Finally, a rather surprising finding of our study was that students who perceived their teachers to have poor leadership skills had significantly less burnout. This contradicts Dahlin, et al.’s study according to which belief in pedagogical shortcomings was a significant predictor of stress [35]. A possible explanation for our result could be that by putting the blame on teachers, such students absolve themselves of responsibility for their academic performance. This could protect them from academic stress. In addition to this, we also found out that students who study a lot and have little time to engage in other activities had low levels of burnout, which at first looks paradoxical but it may be due to students learning strategies to keep themselves motivated to keep up the pace. 

The findings of our study clearly demonstrate that notoriety of medical education for its stressful nature is quite justifiable. Despite this well-known fact, few steps are taken to protect the students from stress and burnout. While reducing syllabus or examinations might decrease the quality of doctors produced and may, thus, be ill-advised, several other steps, including promotion of stress-reduction techniques, can protect medical students from psychological distress [36]. Such strategies could predictably lower burnout among medical students, enabling them to fully concentrate on becoming capable physicians of the future.

The study had a few limitations, which should be considered in future research. The study was conducted in only one city; further research should use a multicentric approach. The cross-sectional design of this study limits deduction about causality and temporality. Students from government and private college were not separately analysed. The English version of CBI was used rather than Urdu, which is the national language of Pakistan.

## Conclusions

Our study reports that burnout is prevalent among the medical students of Pakistan. A lot of medical students and teachers are unaware of the phenomenon. It is of paramount importance that the academic staff should identify this problem, and even if it can't be completely eliminated, its negative effects should be minimised by focusing on the factors we identified to increase the productivity of medical students for better patient care.

## References

[1] Dahlin ME, Runeson B (2015). Burnout and psychiatric morbidity among medical students entering clinical training: a three year prospective questionnaire and interview-based study. BMC Med Educ.

[2] Jadoon NA, Yaqoob R, Raza A, Shehzad MA, Zeshan SC (2010). Anxiety and depression among medical students: a cross-sectional study. J Pak Med Assoc.

[3] Solanky P, Desai B, Kavishwar A, Kantharia SL (2012). Study of psychological stress among undergraduate medical students of government medical college, Surat. Int J Med Sci Public Health.

[4] Carter SB. The Tell Tale (2015). The Tell Tale: Signs of Burnout ... Do You Have Them? (Psychology Today). https://www.psychologytoday.com/blog/high-octane-women/201311/the-tell-tale-signs-burnout-do-you-have-them.

[5] Freudenberger HJ (1974). Staff burn-out. J Soc Issues.

[6] Kaschka WP, Korczak D, Broich K (2011). Burnout a fashionable diagnosis. Dtsch Arztebl Int.

[7] (2015). Occupational burnout. http://https://en.wikipedia.org/wiki/Occupational_burnout.

[8] Dyrbye LN, West CP, Satele D, Boone S, Tan L, Sloan J, Shanafelt TD (2014). Burnout among U.S. medical students, residents, and early career physicians relative to the general U.S. population. Acad Med.

[9] Ishak W, Nikravesh R, Lederer S, Perry R, Ogunyemi D, Bernstein C (2013). Burnout in medical students: A systematic review. Clin Teach.

[10] Cecil J, McHale C, Hart J, Laidlaw A (2014). Behaviour and burnout in medical students. Med Educ Online.

[11] Costa EF, Santos SA, Santos AT, Melo EV, Andrade TM (2012). Burnout Syndrome and associated factors among medical students: a cross-sectional study. Clinics (Sao Paulo).

[12] Backović DV, Zivojinović JI, Maksimović J, Maksimović M (2012). Gender differences in academic stress and burnout among medical students in final years of education. Psychiatr Danub.

[13] Bakker AB, Demerouti E, Schaufeli WB (2002). Validation of the Maslach Burnout Inventory - General Survey: An Internet Study. Anxiety, Stress & Coping.

[14] Dyrbye LN, Thomas MR, Huntington JL, Lawson KL, Novotny PJ, Sloan JA, Shanafelt TD (2006). Personal life events and medical student burnout: a multicenter study. Acad Med.

[15] Dyrbye LN, Thomas MR, Shanafelt TD (2005). Medical student distress: causes, consequences, and proposed solutions. Mayo Clin Proc.

[16] Brazeau CMLR, Schroeder R, Rovi S, Boyd L (2010). Relationships between medical student burnout, empathy, and professionalism climate. Acad Med.

[17] (2015). Copenhagen Burnout Inventory Normative data from a representative Danish population on Personal Burnout Results from the PUMA* study on Personal Burnout, Work Burnout, and Client Burnout. http://goo.gl/kscQuX.

[18] Borritz M, Rugulies R, Bjorner JB, Villadsen E, Mikkelsen OA, Kristensen TS (2006). Burnout among employees in human service work: design and baseline findings of the PUMA study. Scand J Public Health.

[19] Mazurkiewicz R, Korenstein D, Fallar R, Ripp J (2012). The prevalence and correlations of medical student burnout in the pre-clinical years: A cross-sectional study. Psychol Health Med.

[20] Shapiro SL, Schwartz GE, Bonner G (1998). Effects of mindfulness-based stress reduction on medical and premedical students. J Behav Med.

[21] Krasner MS, Epstein RM, Beckman H, Suchman AL, Chapman B, Mooney CJ, Quill TE (2009). Association of an educational program in mindful communication with burnout, empathy, and attitudes among primary care physicians. JAMA.

[22] Shah M, Hasan S, Malik S, Sreeramareddy CT (2015). Perceived stress, sources and severity of stress among medical undergraduates in a Pakistani medical school. BMC Med Educ.

[23] Sreeramareddy CT, Shankar PR, Binu VS, Mukhopadhyay C, Ray B, Menezes RG (2015). Psychological morbidity, sources of stress and coping strategies among undergraduate medical students of Nepal. BMC Med Educ.

[24] Waqas A, Khan S, Sharif W, Khalid U, Ali A (2015). Association of academic stress with sleeping difficulties in medical students of a Pakistani medical school: a cross sectional survey. PeerJ.

[25] Shaikh BT, Kahloon A, Kazmi M, Khalid H, Nawaz K, Khan N, Khan S (2004). Students, stress and coping strategies: a case of Pakistani medical school. Educ Health (Abingdon).

[26] Misra R, McKean M (2000). College students’ academic stress and it’s relation to their anxiety, time management and leisure satisfaction. Am J Health Stud.

[27] Peters M, King J (2012). Perfectionism in doctors. BMJ.

[28] Randolph JJ, Dykman BM (1998). Perceptions of parenting and depression-proneness in the offspring: Dysfunctional attitudes as a mediating mechanism. Cognitive Ther Res.

[29] Soenens B, Elliot AJ, Goossens L, Vansteenkiste M, Luyten P, Duriez B (2005). The intergenerational transmission of perfectionism: parents' psychological control as an intervening variable. J Fam Psychol.

[30] Chang EC, Watkins A, Banks KH (2004). How adaptive and maladaptive perfectionism relate to positive and negative psychological functioning: Testing a stress-mediation model in black and white female college students. J Couns Psychol.

[31] Irvine D (2001). The changing relationship between the public and the medical profession. J R Soc Med.

[32] Zuger A (2004). Dissatisfaction with medical practice. N Engl J Med.

[33] Ahmad W, Krupat E, Asma Y, Fatima N, Attique R, Mahmood U, Waqas A (2015). Attitudes of medical students in Lahore, Pakistan towards the doctor–patient relationship. PeerJ.

[34] Clark EJ, Reiker PP (1986). Gender differences in relationships and stress of medical and law students. J Med Educ.

[35] Dahlin M, Joneborg N, Runeson B (2005). Stress and depression among medical students: a cross-sectional study. Med Educ.

[36] Rosenzweig S, Reibel DK, Greeson JM, Brainard GC, Hojat M (2003). Mindfulness-based stress reduction lowers psychological distress in medical students. Teach Learn Med.

